# Metropolitan Hospital Sunday Fund

**Published:** 1905-08-19

**Authors:** 


### METROPOLITAN HOSPITAL SUNDAY FUND.

### COUNCIL MEETING.

The Question of Aid to Nursing Associations.

The annual meeting of the Council of the Metropolitan Hospital Sunday Fund was held on the 11th inst. at the Mansion House. Sir Sydney] Waterlow, the Vice-President, took the chair in the absence of the President (the Lord Mayor) in connection with the reception of the officers of the French Fleet.

A recommendation of the General Purposes Committee was adopted, raising the salary of the Secretary, Sir Edmund Hay Currie, from £400 to £500 per annum. To give effect to a further recommendation of the General Purposes Committee,

Mr. Carr-Gomm moved that a law be submitted to the next meeting of the constituents of the Fund, providing that the Committee of Distribution should have power to set aside a certain amount as a grant to Queen Victoria's Jubilee Institute for Nurses. He said that the General Purposes Committee had received a deputation from the Jubilee Institute asking that it might be put on the Fund and receive a grant. The Committee heard in detail of the work that was being done, and it was shown that in consequence of having had to spend all the capital they could spend, their income would be reduced in future years. It was pointed out that the work they were doing was cognate with the work of the hospitals; that one of the great troubles was keeping down the out-patient department, and that by the nurses sent out by the institute they were able to administer such relief that numbers were prevented from thronging the outpatient departments and that therefore they were doing a great deal of hospital work in the patients' own homes. The Committee, therefore, felt that it would be a good thing to include the institute in the Fund, but since by the existing laws they could not make them a grant the proposed addition was suggested. As to other nursing societies, it was felt that Queen Victoria's Jubilee Institute was the parent to which all the others were subordinate.

Mr. Richard Biddulph Martin, M.P., seconded the motion, observing that the same thing had been done in the case of the Surgical Aid Society.

Mr. Mackrell said that if they were going to give money to this particular society he did not see why it should be limited to them. They had in South London a nursing association which did as much as the Victoria Institute, and he did not see how they could resist applications from the many other societies equally deserving.

Mr. Norman said he could not see how they were to have a system of analysis of accounts and efficiency in the case of nursing associations as they had with the hospitals, and thought some detailed method of investigation should be arranged before they took this step.

The Rev. E. H. Pearce thought the best way of dealing with the matter would be to refer it back to the General Purposes Committee for further consideration. He moved an amendment to this effect. He quite agreed that nurses ought to be taken into consideration by the Fund, but not one association only. If they decided to do it, let it be known that the Committee was open to consider applications from nursing societies.

The Hon. Sydney Holland agreed that if a grant were made to the Queen Victoria Institute, they need not necessarily make them to others also. No work was better done than that of the nurses who helped to relieve the pressure on the out-patient departments, and no work was more closely allied to that of the hospitals. These nurses were working right in the midst of the poor in the districts all round the hospitals. He did not, therefore, think they need mind other nursing associations applying for aid.

Sir Henry Burdett said he wished to second the proposal to refer the matter back to the General Purposes Committee. He agreed that there was probably no more useful work than that done by the district nurses, and that there was a very close relation between it and the work of the hospitals. It was certain too that they would not get the reform in the out-patient departments which they all desired until they brought together the nursing associations and the hospitals. Then they would have the opportunity of finding out the real circumstances of applicants for relief. As to the South London District Nurses' Association to which reference had been made he could speak with knowledge. The efficiency of the work was remarkable, and the accurate acquaintance of the superintendent with the circumstances of the people was most encouraging. But when they looked at this matter from the point of view of the Fund they must have knowledge to go upon, and must definitely make up their minds as to how far they were going. Otherwise, if they were to take in the Biblewoman type of nurse, who only had three months' training, they would soon be asked to give assistance to every parish nurse also. They wanted more knowledge, and a system carefully thought out and crystallised before they took the step proposed.

The amendment to refer the matter back was then put, and carried by a large majority.

In moving the adoption of the report of the Distribution Committee and the payment of the awards recommended, the Chairman said that since its foundation in 1872 the Fund had distributed no less than £1,382,160. He thought he might say, therefore, that "Hospital Sunday" had been a success. The collections this year, so far, exceeded £75,000, and as the year did not end till October 31st, a considerable increase on that amount might still be expected. Hospital Sunday this year had fallen a fortnight later than usual. There had been a serious decline in some of the larger collections, but there were also many increases, and the sum of £12,500 placed at the disposal of the Lord Mayor had established this year as a record year. (Hear, hear.) Mr. George Herring had allowed the Secretary to state that money collected in the City was to be counted as if collected in the churches, for the purposes of the 5s. in the pound he was good enough to add. They had only had a week for the organisation of that collection, and he hoped another year, if Mr. Herring repeated his generous offer, that they would do better still. There were this year 222 institutions receiving grants, and the amount distributed was over £71,000.

Sir James Ritchie seconded.

Dr. Glover, while congratulating the Council upon the fine results achieved, pointed out that while in the last two reports there had been a reference to the question of expenditure by the hospitals, there was no such allusion this year. He would like to know whether their suggestions were being attended to and if reductions were being effected. It would encourage the public to double their contributions if they were satisfied on that point.

The Chairman assured the meeting that this matter was constantly under the consideration of the Committee. Their influence was used to encourage economy with efficiency, and they were then sending out a circular which he hoped would prove very helpful in that direction.

Mr. Mackrell thought they ought to recognise that a great deal of the increase was due to the exertions of the Secretary and his son. The report was then adopted.

Sir William Church proposed, and Mr. Holland seconded, a vote of thanks to Mr. Herring for his generous and continued interest in the Fund, which was agreed to with acclamation.

Sir Henry Burdett then moved :—

That the thanks of the Council be given to the members of the Committee of Distribution for the care bestowed in the preparation of the awards recommended.

In view of the record collection (£75,000) made that year some of them who had been associated with the Fund for many years must carry their memories back to earlier days. It was well in such circumstances to recall the zeal of the late Dr. James Wakley, who at the time when the collections fell to under £30,000, urged upon the Council and himself that greater efforts must be made until at least £60,000 was raised. It must never be forgotten too that two years before Sir Sydney Waterlow became Lord Mayor and started the Fund at the Mansion House, Dr. James Wakley, as editor of the *Lancet*, came forward and stirred up public interest week after week in Hospital Sunday for the Metropolis. Dr. James Wakley's enthusiasm and interest in hospitals should never be forgotten, nor his initial efforts to bring Hospital Sunday from the provinces to London. It was a good thing to remember those who went before them, to whose inspiration the present success was so largely due. One point in the report he should like to refer to—the statement that, in calculating the cost of out-patients in future, the number of attendances was to be taken instead of the number of cases. He hoped the Committee would continue to require a return of the number of out-patients, for if they sunk that in the number of attendances, there might be a recurrence of the abuses which used to be prevalent in that connection. A return of the actual hospital population was that to which they ought to adhere. Unless they had the correct figures of actual out-patients treated each year, erroneous deductions as to their cost were sure to be made. He also thought that some notice should be taken of the difference in the basis of the cost per bed between those hospitals which provided everything and those which required their patients to supply butter and tea and many other necessaries. These amounted in some cases to as much as £2 per bed, so that a proper comparison could not be made unless this item was taken into account He had the greatest pleasure in proposing thanks to the Committee of Distribution, for it was the one body in which they all had entire confidence.

The resolution was seconded and agreed to; and the Chairman returned thanks, observing that for 33 years the work had been with him a labour of love.

The Rev. A. A. Harland moved a vote of thanks to the newspapers which had pleaded the cause of the Fund, making special reference to the *Lancet* and The Hospital.

This was adopted, and votes of thanks to the Lord Mayor and to the Chairman brought the proceedings to a close.

### DISTRIBUTION COMMITTEE'S REPORT AND AWARDS.

The Committee of Distribution in submitting their report desire to record with regret the resignation of Captain Cundy, who had been an active member of this committee since 1894, and is now unable to give the necessary time to the work of the Fund.

Mr. George Herring has again most liberally offered to add one-fourth to the amount collected in places of worship, and has also generously extended his offer to amounts collected in he City, whether collected in places of worship or not.

The total amount of the Fund to the present time is £61,280, to which has to be added the amount of £12,500 placed at the disposal of the Lord Mayor by the executors of the will of the late Mr. Wyndham Francis Cook, in response to the appeal to City men made on behalf of the Hospital Sunday Fund, making a total of £73,780.

Your committee now recommend the distribution of £71,466 10s. to the 162 hospitals and 60 dispensaries shown in the Appendix attached hereto.

The committee having been unofficially informed that the committee of the City Orthopædic Hospital are prepared to amalgamate with the other two Orthopædic Hospitals, subject o the seven propositions put forward by King Edward's Fund being somewhat modified, this committee therefore recommend a grant to be made subject to the amalgamation being carried through.

The committee desire to impress upon institutions receiving grants from this Fund that awards are made towards the maintenance of patients, and not for building purposes.

The committee recommend that in calculating the cost of out-patients in future, the number of attendances be taken, instead of the number of cases.

This year 223 institutions have made application for grants from the Fund, being five more than in 1904.

One hospital which applied for a grant was considered ineligible.

The committee having reduced the bases of award in 27 cases, this fact was intimated to each hospital interested in accordance with Rule V. Twelve deputations attended the committee, and after hearing their cases, the final awards were determined.

Five per cent. of the total sum collected is set apart to purchase surgical appliances (in obedience to Law XII.) in monthly proportions during the ensuing year.

In compliance with an order of the Council, and for the special use of its members, tables of statistics have been prepared as usual, showing an analysis of the number of beds in hospitals, the cost of patients both at hospitals and dispensaries, the proportionate expense of management, as well as other valuable information, and copies are sent to every member of the Council.

### PARTICULARS OF AWARDS.

Particulars of the Awards recommended by the Committee of Distribution for the year 1905.

### 31 GENERAL HOSPITALS.

Charing Cross Hospital, £1,401; French Hospital, £439; German Hospital, £712; Great Northern Central Hospital, £1,306; Guy's Hospital, £2,398; Hampstead General Hospital, £279; Italian Hospital, £249; King's College Hospital, £1,810; London Hospital, £6,768; London Homœopathic Hospital, £629; Phillips' Memorial Homœopathic Hospital, £42; London Temperance Hospital, £961; Metropolitan Hospital, £1,009; Mildmay Hospital, £356; Miller Hospital and Royal Kent Dispensary, £326; North-West London Hospital, £629; Poplar Hospital, £771; Queen's Jubilee Hospital, £231; Royal Free Hospital, £1,484; St. George's Hospital, £2,291; S.S. John and Elizabeth Hospital, £380; St. John's Hospital, Lewisham, £237; St. Mary's Hospital, £2,980; Seamen's Hospital Society, £1,745; The Middlesex Hospital and Convalescent Home, £3,235; Tottenham Hospital, £712; University College Hospital, £2,084; Walthamstow, &c., Hospital, £148; West Ham Hospital, £676; West London Hospital, £1,246; Westminster Hospital, £1,900.

### SPECIAL HOSPITALS.

#### 5 Chest Hospitals.

City of London Hospital for Diseases of the Chest, Victoria Park, £1,199; Hospital for Consumption, Brompton, £2,612; Mount Vernon Consumption Hospital, Hampstead, £997

Royal Hospital for Diseases of the Chest, City Road, £724; Royal National Hospital for Consumption, Ventnor, £439.

18 Children's Hospitals.

Alexandra Hospital for Hip Disease, W.C., £421; Banstead Surgical Home, £29; Barnet Home Hospital, £53; Belgrave Hospital for Children, S.W., £112; Cheyne Hospital for Incurable Children, S.W., £178; East London Hospital for Children, Shadwell, E., £1,068; Evelina Hospital for Sick Children, Southwark, S.E., £285; Home for Incurable Children, Hampstead, £95; Home for Sick Children, Sydenham, S.E., £154; Hospital for Sick Children, Great Ormond Street, W.C., £1,496; Kensington, for Children and Dispensary, £112; North-Eastern Hospital for Children, Hackney Road, N.E., £415; Paddington Green Hospital for Children, W., £380; St. Mary's Hospital, Plaistow, E., £344; St. Monica's Hospital, Brondesbury, N.W., £83; Victoria Hospital for Children, King's Road, Chelsea, S.W., £575; Victoria Home, Margate, £47; Hospital for Hip Disease, Sevenoaks, £53.

6 Lying-in Hospitals.

British Lying-in Hospital, Endell Street, W.C., £71; City of London Lying-in Hospital, City Road, E.C., £102; Clapham Maternity Hospital, £35; East End Mothers' Home, £95; General Lying-in Hospital, Lambeth, S.E., £65; Queen Charlotte's Lying-in Hospital, Marylebone Road, W., £575.

6 Hospitals for Women.

Chelsea Hospital for Women, S.W., £385; Hospital for Women, Soho Square, W., £445; Grosvenor Hospital for Women and Children, Vincent Square, S.W., £142; New Hospital for Women, Euston Road, W.C., £326; Royal Waterloo Hospital for Children and Women, Lambeth, S.E., £237; Samaritan Free Hospital, Marylebone Road, W., £510.

28 OTHER SPECIAL HOSPITALS.

Cancer Hospital, Brompton, S.W., £118; Middlesex Hospital, Cancer Wing, £333; London Fever Hospital, Islington, N., £356; Gordon Hospital for Fistula, Vauxhall Bridge Road, S.W., £29; St. Mark's Hospital for Fistula, City Road, E.C., £237; National Hospital for the Diseases of the Heart, Soho Square, W., £181; Female Lock Hospital, Harrow Road, W., £298; Hospital for Epilepsy, Paralysis, and other Diseases of the Nervous System, Maida Vale, W., £102; National Hospital for the Paralysed and Epileptic, W.C., £1,436; West End Hospital for Diseases of the Nervous System, £336; Central London Ophthalmic Hospital, Gray's Inn Road, W.C., £198; Royal Eye Hospital, St. George's Circus, S.E., £356; Royal London Ophthalmic Hospital, City Road, E.C., £1,282; Royal Westminster Ophthalmic Hospital, Charing Cross, W.C., £201; Western Ophthalmic Hospital, Marylebone Road, W., £90; City Orthopædic Hospital, Hatton Garden, E.C., £195; National Orthopædic Hospital, Great Portland Street, W., £173; Royal Orthopædic Hospital, Oxford Street, W., £152; Royal Sea Bathing Hospital, Margate, £356; St. John's Hospital for Diseases of the Skin, £35; Western Skin Hospital, Great Portland Street, W., £11; St. Peter's Hospital for Stone, Covent Garden, £89; Central London Throat and Ear Hospital, Gray's Inn Road, W.C., £53; Hospital for Diseases of the Throat, Golden Square, W., £59; London Throat Hospital, Great Portland Street, W., £40; Royal Ear Hospital, Frith Street, W., £32; Royal Dental Hospital of London, W.C., £293; National Dental Hospital, 149 Great Portland Street, W., £27.

32 CONVALESCENT HOSPITALS.

Metropolitan Convalescent Institution, Walton and Broadstairs, £771; Metropolitan Convalescent Institution, Bexhill, £475; All Saints' Convalescent Hospital, Eastbourne, £475; All Saints' Convalescent Home, St. Leonards-on-Sea, £29; Ascot Priory Convalescent Home, £89; Brentwood, for Children, £11; Charing Cross Hospital Convalescent Home, Limpsfield, £100; Chelsea Hospital for Women Convalescent Home, St. Leonards, £47; Deptford Medical Mission Convalescent Home, Bexhill, £29; Mrs. Gladstone Convalescent Home, Mitcham, £109; Friendly Societies' Convalescent Home, Dover, £106; Hahnemann Convalescent Home, Bournemouth, £47; Hanwell Convalescent Home, £23; Hendon, Ossulston Home, £66; Herbert Convalescent Home, Bournemouth, £35; Herne Bay Baldwin-Brown Convalescent Home, £38; Homœopathic Hospital Convalescent Home, Eastbourne, £17; Hemel Hempstead Convalescent Home, £66; Mrs. Kitto's Convalescent Home, Reigate, £59; Mary Wardell Convalescent Home for Scarlet Fever, £100; Morley House Convalescent Home for Working Men, £190; Alfred Bevan Memorial, Sandgate, £261; Police Seaside Home, Brighton, £59; St. Andrew's Convalescent Home, Clewer, £118; St. Andrew's Convalescent Home, Folkestone, £213; St. John's Home for Convalescent and Crippled Children, Brighton, £38; St. Joseph's Convalescent Home, Bournemouth, £71; St. Leonards-on-Sea Convalescent Home for Poor Children, £130; St. Mary's Convalescent Home, Shortlands, £26; St. Michael's Convalescent Home, Westgate-on-Sea, £41; Seaside Convalescent Hospital, Seaford, £142; Fairlight Convalescent Home, Hastings, £23.

23 COTTAGE HOSPITALS.

Acton Cottage Hospital, £71; Beckenham Cottage Hospital, £103; Blackheath and Charlton Cottage Hospital, £106; Bromley, Kent, Cottage Hospital, £148; Bushey Heath Cottage Hospital, £47; Canning Town Cottage Hospital, £64; Chislehurst, Sidcup, and Cray Valley Cottage Hospital, £71; Coldash Cottage Hospital, £26; East Ham Cottage Hospital, £77; Eltham Cottage Hospital, £55; Enfield Cottage Hospital, £90; Epsom and Ewell Cottage Hospital, £72; Hounslow Cottage Hospital, £65; Kingston, Victoria Hospital, £80; Livingstone Cottage Hospital, Dartford, £105; Mildmay Cottage Hospital, £19; Reigate and Redhill Cottage Hospital, £136; Sidcup Cottage Hospital, £35; Tilbury (Passmore Edwards) Cottage Hospital, £59; Willesden Cottage Hospital, £152; Wimbledon Cottage Hospital, £54; Wimbledon South Cottage Hospital, £54; Woolwich and Plumstead Cottage Hospital, £61.

13 INSTITUTIONS.

Bolingbroke Hospital, £296; Hospital for Invalid Gentlewomen, Harley Street, £118; St. Saviour's Hospital and Nursing Home, £119; National Sanatorium for Consumption, Bournemouth, £118; Invalid Asylum, Stoke Newington, £53; Firs Home, Bournemouth, £23; St. Catherine's Home, Ventnor, £29; Friedenheim Hospital for the Dying, £368; Oxygen Home, Fitzroy Square, £39; Santa Claus Home, Highgate, £71; Free Home for Dying, Clapham, £154; St. Luke's House, Pembridge Square, W., £142; Royal Mineral Water Hospital, Bath, £59.

60 DISPENSARIES.

Battersea Provident Dispensary, £130; Billingsgate Medical Mission, £76; Blackfriars Provident Dispensary, £23; Bloomsbury Provident Dispensary, £17; Brixton, etc., Dispensary, £66; Brompton Provident Dispensary, £23; Buxton Street Dispensary, £29; Camberwell Provident Dispensary, £98; Camden Town Provident Dispensary, £19; Chelsea, Brompton, and Belgrave Dispensary, £59; Chelsea Provident Dispensary, £16; Childs Hill Provident Dispensary, £20; City Dispensary, £59; City of London and East London Dispensary, £11; Clapham General and Provident Dispensary, £28; Deptford Medical Mission, £28; Eastern Dispensary, £42; East Dulwich Provident Dispensary, £51; Farringdon General Dispensary, £59; Finsbury Dispensary, £53; Forest Hill Provident Dispensary, £49; Greenwich Provident Dispensary, £39; Hackney Provident Dispensary, £20; Hampstead Provident Dispensary, £71; Holloway and North Islington Dispensary, £59; Islington Dispensary, £74; Islington Medical Mission, £48; Kensal Town Provident Dispensary, £17; Kentish Town Medical Mission, £23; Kilburn, Maida Vale, and St. John's Wood Dispensary, £48; Kilburn Provident Medical Institution, £57; London Dispensary, Spitalfields, £16; London Medical Mission, Endell Street, £128; Margaret Street, for Consumption, £21; Metropolitan Dispensary, £71; Notting Hill Provident Dispensary, £17; Paddington Provident Dispensary, £33; Portland Town Dispensary, £16; Public Dispensary, Clare Market, W.C., £43; Queen Adelaide's Dispensary, £38; Royal General Dispensary, £27; Royal Pimlico Provident Dispensary, £59; Royal South London Dispensary, £61; St. George's and St. James's Dispensary, £64; St. George's, Hanover Square, Provident Dispensary, £35; St. John's Wood Provident Dispensary, £55; St. Marylebone General Dispensary, £55; St. Pancras and Northern Dispensary, £35; St. Pancras Medical Mission, £24; South Lambeth, Stockwell, and North Brixton Dispensary, £47; Stamford Hill, Stoke Newington Dispensary, £71; Tower Hamlets

Dispensary, £54; Walworth Provident Dispensary, £17; Wandsworth Common Provident Dispensary, £16; Westbourne Provident Dispensary, £21; Western Dispensary, £76; Western General Dispensary, £142; Westminster General Dispensary, £52; Whitechapel Provident Dispensary, £41; Woolwich Provident Dispensary, £28.

